# Three Members of Transmembrane-4-Superfamily, TM4SF1, TM4SF4, and TM4SF5, as Emerging Anticancer Molecular Targets against Cancer Phenotypes and Chemoresistance

**DOI:** 10.3390/ph16010110

**Published:** 2023-01-11

**Authors:** Nur Syafiqah Rahim, Yuan Seng Wu, Maw Shin Sim, Appalaraju Velaga, Srinivasa Reddy Bonam, Subash C. B. Gopinath, Vetriselvan Subramaniyan, Ker Woon Choy, Sin-Yeang Teow, Ismail M. Fareez, Chandramathi Samudi, Shamala Devi Sekaran, Mahendran Sekar, Rhanye Mac Guad

**Affiliations:** 1 Department of Pharmaceutical Life Sciences, Faculty of Pharmacy, Universiti Malaya, Kuala Lumpur 50603, Malaysia; 2 Department of Biology, Faculty of Applied Sciences, Universiti Teknologi MARA, Perlis Branch, Arau Campus, Arau 02600, Malaysia; 3 Collaborative Drug Discovery Research (CDDR) Group, Faculty of Pharmacy, Universiti Teknologi MARA (UiTM), Selangor Branch, Puncak Alam Campus, Bandar Puncak Alam 42300, Malaysia; 4 Centre for Virus and Vaccine Research, School of Medical and Life Sciences, Sunway University, Petaling Jaya 47500, Malaysia; 5 Department of Biological Sciences, School of Medical and Life Sciences, Sunway University, Petaling Jaya 47500, Malaysia; 6 Department of Medicinal Chemistry, Faculty of Pharmacy, MAHSA University, Jenjarom 42610, Malaysia; 7 Department of Microbiology and Immunology, University of Texas Medical Branch, 301 University Blvd, Galveston, TX 77555, USA; 8 Faculty of Chemical Engineering & Technology, Universiti Malaysia Perlis (UniMAP), Arau 02600, Malaysia; 9 Institute of Nano Electronic Engineering, Universiti Malaysia Perlis (UniMAP), Kangar 01000, Malaysia; 10 Micro System Technology, Centre of Excellence (CoE), Universiti Malaysia Perlis (UniMAP), Pauh Campus, Arau 02600, Malaysia; 11 Department of Pharmacology, School of Medicine, Faculty of Medicine, Bioscience and Nursing, MAHSA University, Jenjarom 42610, Malaysia; 12 Department of Anatomy, Faculty of Medicine, Universiti Teknologi MARA, Sungai Buloh 47000, Malaysia; 13 Department of Biology, College of Science and Technology, Wenzhou-Kean University, 88 Daxue Road, Quhai, Wenzhou 325060, China; 14 School of Biology, Faculty of Applied Sciences, Universiti Teknologi MARA, Selangor Branch, Shah Alam Campus, 40450 Shah Alam, Malaysia; 15 Department of Medical Microbiology, Faculty of Medicine, Universiti Malaya, Kuala Lumpur 50603, Malaysia; 16 Faculty of Medical and Health Sciences, UCSI University, Kuala Lumpur 56000, Malaysia; 17 Department of Pharmaceutical Chemistry, Faculty of Pharmacy and Health Sciences, Royal College of Medicine Perak, Universiti Kuala Lumpur, Ipoh 30450, Malaysia; 18 Department of Biomedical Science and Therapeutics, Faculty of Medicine and Health Science, Universiti Malaysia Sabah, Kota Kinabalu 88400, Malaysia

**Keywords:** transmembrane 4 superfamily, transmembrane 4 L6 domain family, TM4SF, cancer progression, chemoresistance, targeted cancer therapy

## Abstract

There are six members of the transmembrane 4 superfamily (TM4SF) that have similar topology and sequence homology. Physiologically, they regulate tissue differentiation, signal transduction pathways, cellular activation, proliferation, motility, adhesion, and angiogenesis. Accumulating evidence has demonstrated, among six TM4SF members, the regulatory roles of transmembrane 4 L6 domain family members, particularly TM4SF1, TM4SF4, and TM4SF5, in cancer angiogenesis, progression, and chemoresistance. Hence, targeting derailed TM4SF for cancer therapy has become an emerging research area. As compared to others, this review aimed to present a focused insight and update on the biological roles of TM4SF1, TM4SF4, and TM4SF5 in the progression, metastasis, and chemoresistance of various cancers. Additionally, the mechanistic pathways, diagnostic and prognostic values, and the potential and efficacy of current anti-TM4SF antibody treatment were also deciphered. It also recommended the exploration of other interactive molecules to be implicated in cancer progression and chemoresistance, as well as potential therapeutic agents targeting TM4SF as future perspectives. Generally, these three TM4SF members interact with different integrins and receptors to significantly induce intracellular signaling and regulate the proliferation, migration, and invasion of cancer cells. Intriguingly, gene silencing or anti-TM4SF antibody could reverse their regulatory roles deciphered in different preclinical models. They also have prognostic and diagnostic value as their high expression was detected in clinical tissues and cells of various cancers. Hence, TM4SF1, TM4SF4, and TM4SF5 are promising therapeutic targets for different cancer types preclinically and deserve further investigation.

## 1. Introduction

Uncontrollable proliferation, activated invasion, and metastasis are the key features of cancer progression [1,2]. The mortality and morbidity of cancer patients have a complicated relationship, not only with the early diagnosis but also with preclinical and laboratory research, particularly when attempting to identify and understand the mechanisms involved in cancer progression and prognostic markers [3,4]. Due to the lack of inhibitory responsiveness in cellular mechanisms, the abilities of cancer cells to self-renew, epithelial-to-mesenchymal transition (EMT), and established secondary tumors could lead to a mechanism that promotes cell migration to distant sites [5,6]. Additionally, cancer cells can tremendously alter metabolic pathways to meet their biological need for differentiation and effective proliferation [7,8,9].

Cancer is one of the most lethal diseases worldwide, as more than 19 million cases and 10 million deaths were reported in 2020, and the number is projected to rise dramatically by 2040 [10]. Two-thirds of cancer patients die within several years due to tumor recurrence and metastasis [11]. Therefore, effective cancer diagnosis and targeted treatment are essential to reducing cancer mortality [12]. Chemotherapy, either adjuvant or neoadjuvant treatment, is used to prolong patients’ survival rather than treat the disease [13,14]. In fact, chemotherapy has failed in most cases at the invasion and metastasis phases, which is called cancer chemoresistance. Cancer chemoresistance involves different molecular mechanisms, including transporter pumps, oncogenes, tumor suppressor genes, mitochondrial alteration, deoxyribonucleic acid (DNA) repair, autophagy, EMT, cancer stemness, and exosomes [15,16,17]. Cancer cells have mechanisms that make them chemoresistant, including anticancer drug inactivation, cell death inhibition or apoptosis suppression, drug metabolism alteration, epigenetic change, drug target alteration, target gene amplification, and DNA repair improvement [18,19,20]. Given these, researchers are now focusing on anticancer research at both cellular and molecular levels to improve treatment efficacy and prognosis by controlling tumor recurrence and distant metastasis in cancer patients [21].

Tetraspanins play a role in regulating cell differentiation, migration, proliferation, tumor progression, and chemoresistance [22]. TM4SF is a branch of the tetraspanin superfamily [23], and almost all animal cells contain at least one TM4SF protein [24]. Basically, TM4SF has two divergent extracellular loop domains, the larger of which contains several conserved amino acid motifs, highly conserved hydrophobic tetra-transmembrane domains, and two short cytoplasmic domains at the free amine group (NH2) and carboxyl (COOH) terminals [25]. Several TM4SF members might be involved in cell signaling to modulate tumor progression or metastasis. In contrast, some might be involved in signal transduction pathways and cell activation, development, proliferation, and motility [26].

Generally, six members of TM4SF have been reported with similar topology and sequence homology, including TM4SF1/L6-Ag, TM4SF4/IL-TMP, TM4SF5/L6H, TM4SF18/L6D, TM4SF19/OCTM4, and TM4SF20/TCCE518 [27,28,29]. Among them, TM4SF1, TM4SF4, and TM4SF5 are grouped under the transmembrane 4 L6 domain family [28]. They have been extensively studied on their expression and implicated in various tumor biological activities. For instance, TM4SF1 overexpression has been identified in many cancers, including lung, breast, colon, ovarian, prostate, pancreatic, renal, and glioma [27,28,30,31,32,33,34]. For TM4SF4, its increased levels have been detected in both non-dividing epithelial intestinal cells and hepatocytes responsible for cellular differentiation and migration [35,36,37]. It also plays a critical role in regulating radiotherapy resistance in lung cancer (LC) cells. Moreover, according to Huang, Wang [32], TM4SF4 may affect colorectal cancer (CRC) cells’ metastatic behavior, where its overexpression and *S100B* and *OLFM4* genes have been found in the circulating tumor cells of blood specimens of 103 preoperative CRC patients. Like TM4SF1, the expression profile of TM4SF5 has been studied and highly expressed in many cancers, such as pancreatic, gastric, colon, liver, papilla vateri, soft tissue, non-endocrine lung, and adrenocorticotropic hormone (ACTH)-negative bronchial carcinoid [38,39,40].

In recent years, increasing studies have demonstrated the critical roles of the tetraspanin L6 domain family or TM4SF members in cancer prognosis, leading to their potential consideration as an anticancer molecular target. This paper presents a detailed and updated review of the regulatory roles of TM4SF1, TM4SF4, and TM4SF5 in the progression and chemoresistance of different cancer types preclinically in which their individual impacts on each cancer are described. Furthermore, it reveals the associated molecular mechanisms in promoting their regulatory roles, as well as the key molecules involved. It also discusses the current use of antibody treatment in the research setting to suppress the expression of TM4SF1, TM4SF4, and TM4SF5 and their associated effects on cancer processes.

## 2. Transmembrane 4 Superfamily

**Figure 1:**
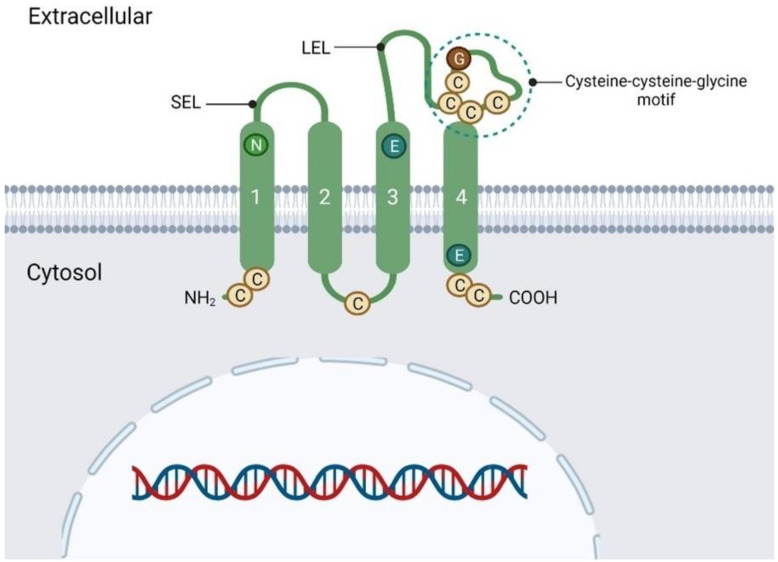
Structure of transmembrane 4 L6 domain.

TM4SF proteins are found in almost all multicellular eukaryotes [41,42]. It consists of four transmembrane alpha (α)-helices and two extracellular domains with one shorter (small extracellular domain/loop, SED/SEL or EC1) and one larger (large extracellular domain/loop, LED/LEL or EC) [29,41], with the structure, is shown in Figure 1. They are differentiated by conserved amino acid sequences, including a CCG motif and other cysteine residues in the EC2 [29,43]. It has been reported that TM4SF can be found in almost all types of mammalian cells and tissues [44]. They can interact with each other and with integrins or receptor-like growth factor receptors to form protein-protein complexes. The interactions further induce intracellular signaling to regulate cell differentiation, activation, growth, and migration [45,46]. By having these properties, TM4SF proteins work as a facilitating factor, as its essential feature is to establish associations with a variety of partner proteins to constitute a tetraspanin web. Thus, TM4SF protein can be associated with other tetraspanins and the members of other families, including immunoglobulins (Ig), integrins, growth factors, and signaling enzymes. Therefore, the main role of TM4SF proteins is to facilitate signal transduction by organizing other proteins [47].

There are six TM4SF members with similar topology and sequence homology, namely TM4SF1/TAAL6, TM4SF4/IL-TMP, TM4SF5/L6H, TM4SF18/L6D, TM4SF19/OCTM4, and TM4SF20/TCCE518. All have similar structures and are involved in cellular processes, such as migration and invasion [48,49]. Among them, TM4SF1, TM4SF4, and TM4SF5 are grouped under the transmembrane 4 L6 domain family [28], and they have been studied for their expression and roles in various tumor biological activities.

In the past decade, TM4SF1 was demonstrated to be expressed and involved in the progression of different cancers, including prostate cancer (PRC) [27], pancreatic cancer (PC) [31], ovarian cancer (OC), breast cancer (BC) [13,50], CRC, and gastric cancer (GC) [51,52]. Comparatively, only several studies have reported the regulatory roles of TM4SF4 in cancers, particularly involved in the cancer prognosis of CRC [32], LC [53], and liver cancer [54]. On the other hand, most studies have confirmed the expression and involvement of TM4SF5 in hepatocellular carcinoma (HCC) [55,56], CRC [57], and PC [58].

This review explicitly discusses the expression profile as diagnostic and prognostic values, together with the regulatory roles and associated molecular mechanisms of TM4SF1, TM4SF4, and TM4SF5 in different cancers, to evaluate its potential as an anticancer therapeutic target, particularly for cancer progression and chemoresistance.

## 3. The Regulatory Roles of TM4SF1 in Different Cancer Types

**Figure 2:**
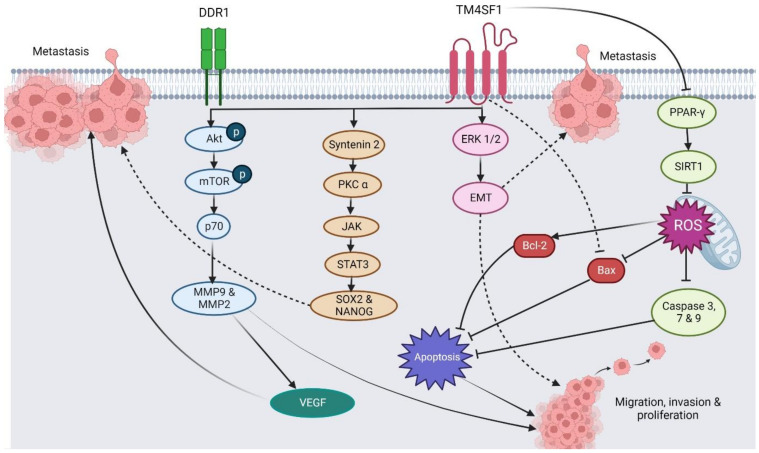
The proposed mechanism underlying the roles of TM4SF1 toward cancer phenotypes and progression.

TM4SF1, known as the tumor-associated antigen L6 (TAAL6), is highly expressed in human epithelial cancers such as BC, OC, CRC, PRC, and PC [13,28,59,60,61,62]. TM4SF1 was first cloned and identified by Marken in 1992 [63,64]. It is a glycoprotein on chromosome 3, with a molecular weight between 21 kDa and 28 kDa [65]. As a distant relative of the TM4SF family due to differed sequence homology, TM4SF1 interacts with other members and other proteins or integrins to mediate different downstream mechanisms [66,67,68]. The activities that TM4SF1 initiates include promoting cell proliferation, cell migration, cell invasion, and forming vascular endothelial pseudopodia or nanopodia [50,62,68,69]. The last decade has seen an increased exploration into the roles of TM4SF1 to further understand its functions in cancer progression and associated molecular mechanisms. Section 3.1, Section 3.2, Section 3.3, Section 3.4, Section 3.5 and Section 3.6 outline the regulatory roles of TM4SF1 in the progression and phenotype of various cancers, with the data summarized in Table 1. The summative associated molecular mechanisms are depicted in Figure 2.

### 3.1. Prostate Cancer

PRC is the second most commonly diagnosed cancer in men worldwide [78]. For patients with metastatic PRC, androgen deprivation therapy (ADT) has been the gold standard [79]. Androgens control the proliferation and survival of prostate epithelial cells by binding to the AR, which is also involved in the development of PRC and regulates specific target genes [80,81]. Both mRNA and protein levels of TM4SF1 are significantly higher in human PRC cells (i.e., PC-3, DU145, LNCaP, and VCaP) as compared to BPH specimens and non-metastasis-derived 22RV1 cells [27]. Buhler et al. [82] observed downregulation of *TM4SF1* gene expression in LNCaP cells after treating with bicalutamide (nonsteroidal anti-androgen) or finasteride (5α-reductase inhibitor), where 5α-reductase catalyzes the conversion of testosterone to dihydrotestosterone (AR agonist) in prostate cells [70]. The data obtained in the study suggested that TM4SF1 could serve as a targeted regulated gene or protein via the interaction between androgen and its receptor. Furthermore, TM4SF1 was confirmed as a direct target gene in a comparative transcriptomic analysis. The result showed an upregulation in the mRNA levels of *TM4SF1* in LNCaP cancer cells treated with androgen in the presence of cycloheximide (CHX, a protein synthesis inhibitor) [27]. CHX was used in the model to inhibit subsequent secondary transcription cascades, allowing the identification of genes that are only directly induced by AR and expressed without intermediate transcription factors. In PRC progression, the AR pathway activation leads to tumor overgrowth; thus, TM4SF1 could be a target gene or potential biomarker for PRC in therapeutic strategies. Additionally, the inhibition of TM4SF1 protein expression significantly reduced cell motility or migration, as assessed in a wound-healing assay [27].

The inhibition of TM4SF1 protein expression due to RNA interference significantly revoked the migration ability of metastatic PC-3 and non-prostatic HeLa cancer cells [71]. In addition, high expression of TM4SF1 in PRC significantly activated the ERK1/2 signaling pathway, increased EMT-related protein (e.g., E-cadherin, V-cadherin, and Vimentin), and enhanced invasion, migration, and proliferation of PRC cells [72]. TM4SF1, highly expressed in PC3 cells, was implanted subcutaneously in nude mice along with a mixture of human ECFC/MSC cells. Its implantation greatly increased the vascularity of Matrigel plugs in mice [59]. The mRNA levels of *TM4SF1* in prostate tumor samples were significantly higher than in non-cancerous prostate glands from patients with BPH. However, no correlation was found in the tumor tissues between mRNA level and Gleason score (PRC grading system) [83], the pathological stage, or the presence of extra-capsular invasion [27]. These findings suggest that TM4SF1 is essential for PRC progression and could be a potential therapeutic target to combat PRC.

### 3.2. Pancreatic Cancer

PC is the world’s fourth leading cause of cancer-related deaths [9]. Cao, Yang [31] observed high expression of TM4SF1 protein in pancreatic tumor tissues as compared to normal pancreatic tissues and chronic pancreatitis tissues. Similarly, the mRNA expression of *TM4SF1* in PC cells was also elevated to a significantly higher level than in human pancreatic duct epithelial cells. However, TM4SF1 did not affect cancer cell proliferation, as examined in vitro and in vivo studies. Nonetheless, *TM4SF1* silencing significantly reduced the invasion and metastasis of PC cells in vivo [31]. MMPs, which are essential for cancer cell migration and invasion, can degrade different types of extracellular matrix (ECM) and affect VEGF-mediated vasculature and metastasis development [9]. Meanwhile, it also downregulated the expression and suppressed the activity of MMP-2 and MMP-9, which have been identified as PC biomarkers. However, no changes in cell proliferation were observed after *TM4SF1* silencing. Moreover, *TM4SF1* silencing also decreased the formation of lung and liver metastasis in orthotopic PC models [31].

In addition, DDR1 (a subfamily of receptor tyrosine kinases), which functions as a cell surface receptor for cancer cell adhesion, proliferation and differentiation, and migration and invasion, has also been associated with TM4SF1-mediated activities. Notably, *TM4SF1* silencing decreased DDR1 expression and induced colocalization with DDR1 in PANC-1 and AsPC-1 cells. Thus, invadopodia formation induced by TM4SF1 in PC cells was associated with DDR1 expression [67]. Additionally, it has been reported that TM4SF1 expression in PC cells can be regulated by microRNAs. For instance, has-miR-141 was reported to inhibit TM4SF1 expression post-transcriptionally by directly targeting the binding site in its three-prime untranslated (3′UTR) region; thus, PC cell migration and invasion were attenuated. However, inhibitory effects were not detected on the cell cycle, proliferation, and apoptosis [49]. In clinical studies, *TM4SF1* mRNA levels were higher in PC tissues, and the expression was positively correlated with DDR1 mRNA expression [67], where DDR1 expression has been linked to a poor prognosis in patients with shorter survival times [84]. In contrast, Zheng, Ohuchida [30] found that patients with low levels of TM4SF1 in PC tissues had higher tumor grade, advanced clinical stages, and shorter survival periods than patients with higher levels of TM4SF1. The local spread was also more common in the group with low TM4SF1 expression. This paradox is most likely due to the fact that they only used resected pancreatic ductal adenocarcinoma (PDAC) tissue. In contrast, the other investigators included PC tissues and paired adjacent tissue in their study.

Overall, the expression of TM4SF1 in PC has a role and correlates to cell invasion, metastasis, and migration via the interactions with DDR1, signaling pathways, transcription factors, and miRNAs. However, it is not involved in cell proliferation, cell cycle, and apoptosis. However, using TM4SF1 as a potential prognostic marker should be approached with caution, considering the specific type of pancreatic cancer.

### 3.3. Gastric Cancer

Due to late diagnosis, GC is one of the gastrointestinal (GI) cancers that contribute to a high mortality rate [9]. High TM4SF1 expression in GC has been previously reported [85]. In contrast to PC, TM4SF1 can regulate human GC cell proliferation. Intriguingly, TM4SF1 upregulation promoted GC MGC803 and MKN45 cell proliferation, which was reversed after gene silencing [52]. Similarly, MGC803 and MKN45 cell invasion and migration were also significantly reduced following *TM4SF1* silencing [52]. The results further indicated that *TM4SF1* silencing significantly decreased the mRNA levels of *Bcl2* while simultaneously upregulating *caspase-3* and *Bax* expression, indicating that TM4SF1 downregulation could promote apoptosis in MGC803 and MKN45 cells [52]. However, these findings are found contradictory to those reported in a clinical study using human patient specimens (gastric mucosa tissues) in which a low TM4SF1 expression was associated with carcinogenesis, progression, and invasion of the tumor, together with poor OS in GC patients [73,74]. Therefore, the promoting role of TM4SF1 in GC remains controversial and deserves more investigations.

### 3.4. Breast Cancer

Being the most prevalent cancer with a high incidence rate worldwide, BC poses a severe threat to women’s health [86,87]. Like other cancer types described above, TM4SF1 was also overexpressed in BC [88]. TM4SF1 overexpression significantly promoted human BC MDA-MB-231 cell migration while suppressing apoptosis [50]. The transient *TM4SF1* silencing using a small interfering RNA (siRNA)-mediated technique significantly reduced cell migration. In contrast, increased cell migration was observed following *TM4SF1* upregulation when transfected with pcDNA-*TM4SF1*. It has been reported that cell migration is mediated by the activation of cell signaling [89]. Thus, *TM4SF1* upregulation could significantly increase the protein expression of p-mTOR, p-P70, and p-AKT. When the *TM4SF1* gene was silenced, its mRNA expression was significantly reduced and inhibited cell migration [50]. Additionally, TM4SF1 has been claimed as a potent mediator for metastatic reactivation of BC through its non-canonical interaction with collagen receptor tyrosine kinase DDR1 via syntenin 2, PKCα, JAK2, and STAT3 signaling pathways in syngeneic BALB/c mice model. Consequently, this activated mechanism induced SOX2 and NANOG expression and led to metastatic reactivation in the lung, bone, and brain [75]. High TM4SF1 expression was also associated with low ER, low PR, and HER2 expression, which are called TNBC. These patients were likely to have shorter DFS and OS [76]. Generally, high TM4SF1 in BC is associated with increased cell migration and metastasis through the activation of cell signaling, together with reduced apoptosis activity and poor survival clinically.

### 3.5. Ovarian Cancer

Aside from BC, OC is another common malignant tumor of the female reproductive organs with a high mortality rate [90]. Gao et al. [13] recently reported the role of TM4SF1 in OC. Intriguingly, TM4SF1 had a higher expression in OC epithelial tissues than in benign ovarian tumor tissues and normal ovarian epithelial tissues. The study further identified that TM4SF1 protein expression was significantly higher in the late stage than in the early stage of OC. Furthermore, *TM4SF1* silencing distinctly suppressed OC cell migration and invasion (e.g., HO8910PM and SKOV3), as well as xenograft tumor growth in nude mice, but that did not affect cell proliferation, cell growth, or cell cycle [13]. Based on the current finding, TM4SF1 could bring new insights into its impact on cancer metastasis. The related mechanisms underlying the involvement of TM4SF1 in OC cell invasion and metastasis deserve further investigation. Given the crucial role of the interplay between TM4SF1 and DDR1, as described above, demolishing the interaction between these two proteins might be vital in finding a potential therapeutic molecular target for OC. However, more in vitro and in vivo studies are essential to prove this assumption.

### 3.6. Hepatocellular Carcinoma

Effective therapies and targeted molecular drugs against HCC are still lacking, with the prognosis of HCC patients remaining poor [91]. Additionally, patients are usually diagnosed with HCC at the advanced stages. In clinical HCC samples, the *TM4SF1* gene was expressed much higher than those in normal cases [92]; thus, it was suggested as one of the risk factors for HCC patient survival [93]. However, Shao, Sun [94] claimed that TM4SF1 was not remarkably associated with the OS time of HCC patients. Again, the controversial finding is most likely due to the different types of HCC samples chosen in both studies. The number of samples tested by Shao, Sun [94] was limited to three cases, only focusing on poor histological differentiation of HCC clinical tumor tissues. The other researcher chose a sample from the Gene Expression Omnibus based on hypoxia-treated HCC cells. Therefore, the type of sample chosen is crucial in comparing the effect of TM4SF1 as a potential biomarker in HCC.

TM4SF1 overexpression in human HCC cells has also been reported [95]. For instance, Huang et al. [69] showed that TM4SF1 overexpression could promote the proliferation, invasion, and metastasis of HCC cells. Furthermore, they also reported that high TM4SF1 expression decreased apoptosis and reciprocally increased the migratory capacity of HCC HepG2 cells by regulating the related genes, including *caspase-3*, *caspase-9*, *MMP-2*, *MMP-9,* and *VEGF*. TM4SF1 also could increase tumor growth and metastasis in a murine model in which these effects were reversed after *TM4SF1* silencing [96]. These findings suggested that TM4SF1 is associated with HCC growth and progression and thus could potentially be a therapeutic target for HCC.

Additionally, TM4SF1 also plays a crucial role in angiogenesis, as its high expression levels have been detected in vascular endothelium cells of human cancers [66,97]. When the *TM4SF1* gene was silenced, human umbilical vein endothelial cells (HUVEC) became immobile, thus indicating that TM4SF1 interacts with integrins to mediate HUVEC migration and enhances intercellular communication [66]. Moreover, Zukauskas et al. [68] demonstrated that TM4SF1 promoted cell migration by increasing filopodia formation. Collectively, high TM4SF1 expression found in HCC cells and tumor-associated HUVEC allude to its potential as a dual therapeutic target.

### 3.7. Bladder Cancer

Compared to other cancer types, one study investigated the biological effects of TM4SF1 on BCa [77]. It was found that TM4SF1 was highly expressed in human muscle-invasive BCa (MIBC) tissues, which significantly correlated with T stage, TNM stage, lymph node metastasis status, and a lower OS rate. These findings indicated that high levels of TM4SF1 in tumor specimens might predispose a high risk of having BCa and a poor prognosis for BCa patients [77]. Additionally, Cao, Wang [77] also reported that *TM4SF1* silencing using shRNA in vitro and in vivo distinctively inhibited cell and tissue proliferation and tumor growth.

ROS are a diverse group of molecules that affect cell components and cellular processes, resulting in pro- and anti-cancer effects. A therapeutic approach that either eliminates or produces ROS could be effective in cancer treatment [98]. For instance, downregulation of *TM4SF1* through siRNA silencing could induce cell cycle arrest, ROS generation, catalase, and SOD2 upregulation, leading to oxidative stress-induced apoptosis. A PPARγ-SIRT1 negative feedback loop may mediate these events. Collectively, this study implied that TM4SF1 has diagnostic and prognostic values for BCa patients and could impact BCa cell growth and proliferation by regulating oxidative stress and PPARγ-SIRT1 [77].

## 4. Molecular Expression, Regulatory Roles, Mechanisms, and Biomolecular Interactions of TM4SF4 in Different Cancers

TM4SF4, also known as the intestine and liver tetraspan membrane protein (ILTMP), was named after the initial cloning of the intestinal epithelium and liver [35,36]. It is a membrane protein that contains four hydrophobic transmembrane domains and two hydrophilic regions. It is classified as a more divergent TM4SF member and lacks the characteristic cysteine residue motifs in the EC2 extracellular domain. Additionally, it has a 50% sequence identity with TM4SF1 [28]. TM4SF4 expression has been observed in several tissues, including the liver, pancreas, and intestinal bulb domains. Its level is increased during the differentiation of non-dividing epithelial cells and the migration of intestinal crypts [99].

**Figure 3:**
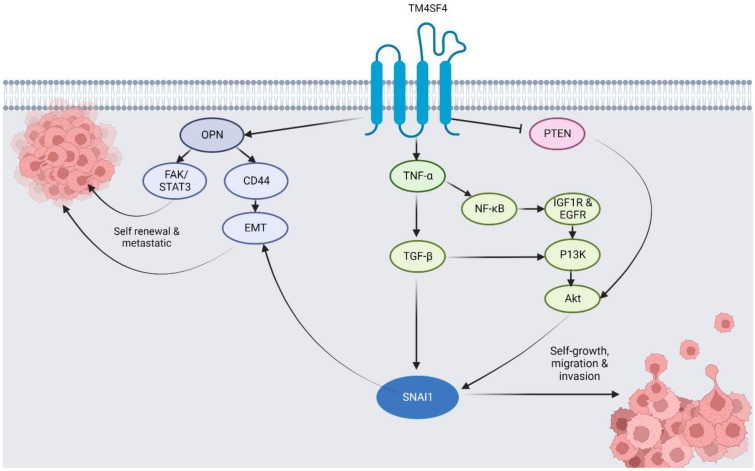
The proposed mechanism underlying the roles of TM4SF4 toward cancer phenotypes and progression.

Compared to TM4SF1, relatively fewer studies have investigated the regulatory roles of TM4SF4 in cancer. However, the findings have shown that TM4SF4 plays an important regulatory role in cancer phenotypes and progression, making it a potential therapeutic molecular target for different cancer types, as described below. The regulatory roles of TM4SF4 in the progression and phenotype of different cancer are discussed in Section 4.1, Section 4.2 and Section 4.3 (Table 2), with the summative associated molecular mechanisms depicted in Figure 3.

### 4.1. Lung Cancer

The tumorigenic activity of TM4SF4 in lung adenocarcinoma was confirmed by immunohistochemical staining of the xenograft model in athymic BALB/c nude mice [48]. As reported by You and Gao [101], *TM4SF4* was identified as one of the top five genes that were significantly expressed in patients with alectinib-resistant LC and was strongly linked to nuclear division, mitosis, and cell cycle. In addition, *TM4SF4* mRNA was sorted as an outlier gene from the CCLE and TCGA data with high absolute expression levels relative to other genes. Furthermore, tissue microarray analysis revealed that five of the 119 lung adenocarcinoma cases scored high TM4SF4 expression. However, as the outlier sample sizes were too small, it may be difficult to determine the relevance of clinicopathological features based on TM4SF4 expression [100].

In LC, TM4SF4 was found to be highly expressed in HCC-1833, Calu-3, and A549 LC cells. The cell cycle and cell growth were inhibited when TM4SF4 was silenced using siRNA or shRNA [100]. Another two studies under the same research team also investigated the regulatory roles of TM4SF4 in LC cells [48,53]. Choi, Kim [48] discovered high TM4SF4 expression in radiation-resistant LC A549 and Calu-3 cells. Interestingly, TM4SF4 overexpression could activate their growth, migration, and invasion. Additionally, the downstream proteins involved in the cell phenotype-related pathways, such as PI3K, AKT, and NF-κB, as well as PTEN, were also up-regulated in A549 cells. TM4SF4 overexpression also activated IGF1R. However, only a slightly higher expression was observed for EGFR. Choi et al. [53] further revealed that TM4SF4 overexpression in A549 cells could increase OPN secretion, which then activated CD44 or integrin signaling, thus maintaining EMT-associated CSC properties. Moreover, it was found that OPN upregulation in an autocrine manner enhanced the incessant self-renewal and metastatic ability of LC cells (A549) via TM4SF4-mediated FAK/STAT3 signaling pathway.

To sum up, TM4SF4 overexpression in LC significantly increases cell growth, migration, and metastasis via interactions with different signaling pathways and growth factors.

### 4.2. Colorectal Cancer

Patients with colorectal liver metastases have a poor prognosis, with a median survival time of 8 months without treatment and 5-year survival rates of 15–50 percent [104]. The promoting role of TM4SF4 in liver metastasis was detected in CRC patients, where the *TM4SF4* gene was upregulated in CRC tissue specimens as compared to normal CRC adjacent tissues. Additionally, the results demonstrated that liver metastasis of CRC was significantly associated with TM4SF4 overexpression, indicating the potential role of TM4SF4 as a biomarker for the advanced stage of CRC [32]. However, the mechanism involved in TM4SF4-mediated liver metastasis of CRC has not been fully elucidated [105]. Furthermore, Li et al. [102] also reported a correlation between the poor prognosis of CRC patients and TM4SF4 overexpression. It has been shown that tumor cells undergo EMT to acquire the characteristics of motility and invasiveness, which are important for them to migrate and invade [106,107]. In this regard, Li et al. [102] also showed that EMT biomarkers, such as TGF-β, TNF-α, and NF-κB, as well as the transcriptional factors (e.g., Snail and PI3K), were involved in the integrated pathways (e.g., TGF-β/Snail or TNF-α/ NF-κB) of TM4SF4 to facilitate EMT process and CRC prognosis. They further found that high TM4SF4 expression predicted poorer CRC prognosis when it was mapped to the EMT-related TGFβ/Snail and TNFα/NFκB pathways and turned out to be correlated with low OS of patients with CRC. For the first time, TM4SF4 was confirmed to affect the prognosis of CRC patients; however, its biological functions are mostly unknown and deserve further investigation [102].

### 4.3. Hepatocellular Carcinoma

Unlike the GI tract studies described above, the potential oncogenic function of TM4SF4 in HCC has not yet been explored much [103]. TM4SF4 protein was found to be highly expressed in HCC cell lines (i.e., QGY-7701, SMMC-7721, BEL-7404, BEL-7404, HepG2, Huh-7, and Hep3B), and low expression was found in the normal liver cell line (i.e., QSG-7701, Chang, and L02). Additionally, TM4SF4 mRNA and protein levels were highly expressed in tumor specimens of HCC patients as compared to non-tumor tissues [54,103], with more abundant in the plasma membrane and less in the cytoplasm and none in the nucleus [54]. According to the immunohistochemical survey of HCC tissue microarray, the expression level of TM4SF4 protein in HCC was linked to tumor progression, as higher expression occurred in the early stages of HCC [103]. Li, Wang [103] reported that overexpression of TM4SF4 in HCC promoted cell proliferation and colony formation (QGY-7701 and BEL-7404), and its reduced level may reverse the observed effects. A study found that TM4SF4 played a crucial role in the proliferation and development of HCC [54]. In the study, when the *TM4SF4* gene was silenced using adenovirus-delivered siRNA targeting TM4SF4 (AdSiTM4SF4) in QGY-7701 and SMMC-7721 HCC cells, the endogenous TM4SF4 expression was significantly inhibited, consequently effectively reducing cell growth and colony formation. Moreover, AdSiTM4SF4 injection into xenograft nude mice significantly suppressed tumor growth and size [54]. Overall, TM4SF4 is implicated in regulating HCC cell proliferation and development. However, additional investigations are required to confirm its impact on HCC phenotypes and associated molecular mechanisms.

## 5. Molecular Expression, Regulatory Roles, Mechanisms, and Biomolecular Interactions of TM4SF5 in Different Cancers

**Figure 4:**
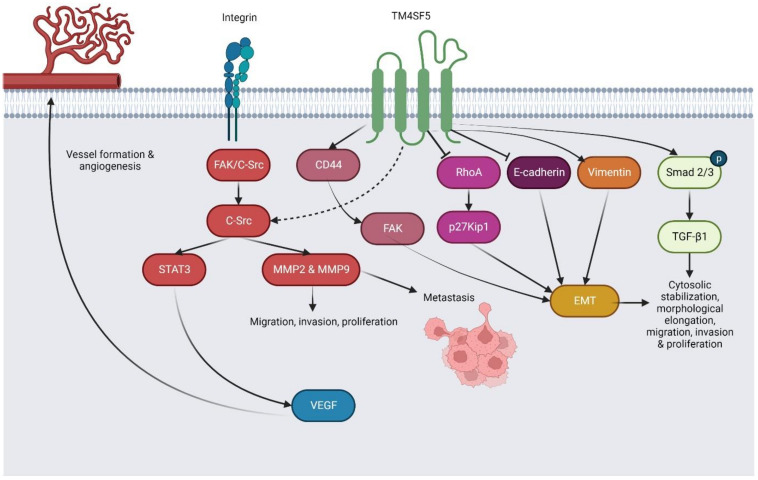
The proposed mechanism underlying the roles of TM4SF5 toward cancer phenotypes and progression.

TM4SF5, also known as IL-TMP, is a transmembrane glycoprotein of TM4SF [108]. *TM4SF5* gene locates on human chromosome 17 and encodes for TM4SF5 protein with a 97 amino acid sequence. It comprises two cysteine residues and two N-glycosylation sites within extracellular loops [28,109]. TM4SF5 has been detected to be highly expressed in esophageal cancer (EC) [110], HCC [111], and CRC [57]. The summative molecular mechanisms underlying TM4SF5 biological activities in cancer phenotypes and progression are described in Figure 4. The expression, regulatory roles, and interactions of TM4SF5 in different cancers, as well as associated molecular mechanisms with the data, are presented in Section 5.1, Section 5.2 and Section 5.3 and Table 3.

### 5.1. Hepatocellular Carcinoma

As detected in human HCC cells and clinical samples, TM4SF5 enhanced cytosolic stabilization and morphological elongation by increasing the expression of p27^Kip1^ (a CDK inhibitor/tumor suppressor) via the modulation of RhoA [38,112]. RhoA is a small GTPase protein in the Rho family of GTPases that is encoded by the *RhoA* gene [122,123]. It is primarily involved in cytoskeletal regulation, which is important for cell progression, metastasis, and cell division, and regulating morphology during apoptosis [124]. Upregulation of both mRNA and protein levels of *RhoA* in HCC tissues has been associated with poor prognosis [125]. Clinically, Cyclin D1 levels were higher in TM4SF5-positive HCC patient tissues, whereas p16 and p18 CDK inhibitors were lower. There was no correlation between EGFR phosphorylation, pErk1/2, β-catenin, or myc levels with cyclin D1 expression [112]. Additionally, *TM4SF5* silencing abolished the expression of p27^Kip1^ and reconstituted E-cadherin, which is involved in the EMT process in a Snail1-independent manner [38].

Furthermore, the ectopic TM4SF5 expression increased FAK Tyr577 phosphorylation associated with FAK, Rho GTPase-activating protein, and c-Src phosphorylation, causing protein RhoA inactivation [38,108]. It has been demonstrated that treatments with 4’-(p-toluenesulfonylamido)-4-hydroxychalcone (TSAHC) or its derivatives could decrease pY577FAK and p27^kip1^ levels in HCC cells [113]. Primarily, TM4SF5-mediated RhoA inactivation promoted EMT, leading to tumor cell migration, invasion, and proliferation due to the loss of contact inhibition [112].

In addition to mediating EMT [38,115], TM4SF5 also induced self-renewal and other properties of circulating tumor cells via the interaction with CD44 [108,114]. It directly binds to and activates FAK in an adhesion-dependent manner and thus activates HCC migration and invasion [126]. Apart from the above findings, TM4SF5 also plays a vital role in angiogenesis. For instance, TM4SF5 overexpression was also correlated with VEGF expression and vessel formation in HCC SNU449 cells and clinical HCC samples [115]. Additionally, TM4SF5-expressing cells stimulated the tube formation of primary HUVEC and the outgrowth of endothelial cells from the aorta ring segments. The anti-VEGF antibody significantly abolished these effects. More importantly, the above-mentioned TM4SF5-mediated effects required the regulation of integrin-α5, c-Src, and STAT3 [108,115].

In another study, TM4SF5 overexpression in nude mice facilitated migration, invadopodia formation, MMP activation, invasion, and eventually lung metastasis, with the reverse effects observed after *TM4SF5* silencing using shRNA [116]. Lee, Kim [116] revealed that TM4SF5 efficiently mediated the formation of invadopodia to degrade ECM during the invasion, as known invadopodia markers (e.g., Actin Related Protein 2 complex (Arp2), N-WASP, cortactin, and MT1-MMP) were located at actin-enriched invadopodia in TM4SF5-expressing cells. They also revealed the role of TM4SF5 in causing uncontrolled growth of human HCC cells through EMT. High TM4SF5 expression in HCC cell line and clinical samples was correlated with p27^Kip1^ upregulation, cytosolic stabilization, and morphological elongation mediated by RhoA inactivation, resulting in EMT induction via the loss of E-cadherin expression. Consequently, aberrant cell growth, anchorage-independent growth, and tumor formation in nude mice were observed. Additionally, they also reported that TM4SF5 could transduce intracellular signaling to activate FAK/c-Src, MMP2, and MMP9, which played important roles in TM4SF5-enhanced invasion and was further correlated with an enhanced metastasis to mouse lung. Tumor tissue from HCC patients had higher TM4SF5 and Smad2/3 phosphorylation levels, implying a possible link between TGFβ1 signaling and TM4SF5 expression in liver carcinogenesis [117]. Therefore, the regulatory roles of TM4SF5 in HCC metastasis are promising and thus can be a therapeutic molecular target for it.

### 5.2. Esophageal Cancer

EC is the world’s seventh most common cancer and the sixth leading cause of cancer death [127]. Wu et al. [110] revealed that high TM4SF5 expression was detected in EC cells and clinical tissue samples as compared to normal human esophageal epithelial cells and non-tumor tissues. The five-year OS of EC patients with TM4SF5high/integrin β1 high after the surgical operation was higher than patients with TM4SF5low/integrin β1low.

The proliferation of EC KYSE150 cells was remarkably reduced in 3-[4,5-dimethylthiazole-2-yl]-2,5-diphenyltetrazolium bromide (MTT) assay following *TM4SF5* downregulation. Wound closure of *TM4SF5* knockdown was markedly delayed in the wound-healing assay, indicating that the gene promotes KYSE150 metastasis [110]. The formation of the TM4SF5-integrin β1 complex inhibited laminin 5-mediated cell invasion, indicating that the combination of TM4SF5-integrin β1 can be a potential clinical target in EC prognosis [23,110]. Collectively, TM4SF5 is implicated in EC cell proliferation, metastasis, and invasion via interactions with integrin.

### 5.3. Pancreatic Cancer, Colorectal Cancer, and Gastric Cancer

In addition to the cancer types mentioned above, TM4SF5 was also highly expressed in a mouse allograft model of PC cells and human PC tissues as compared to normal pancreatic tissue [39,55,58]. Large-scale screening of differential gene expression involved in the carcinogenesis of the pancreas was performed using a radiation hybrid panel, leading to the identification of TM4SF5 as the overexpressed gene that is highly homologous to tumor-associated antigen L6 [39]. Intriguingly, Park, Kim [58] found that TM4SF5-transfected PANC02 cells markedly increased cell proliferation and motility, as well as the growth of tumor mass in mice [58].

TM4SF5 expression has been detected in a mouse CRC cell line (CT-26) [118]. *TM4SF5* silencing in CRC LoVo and SW480 cells significantly mitigated their proliferation by showing low absorbance in Cell Counting Kit*-*8 *(*CCK-8), a cell viability assay. The wound-healing assay proved that TM4SF5-sh of both cells had reduced cell migration and invasive ability in transwell assay [119]. Using a specialized antibody, it recognized a recombinant TM4SF5 was overexpressed in CRC cells and human CRC tissues. Moreover, high TM4SF5 expression was also significantly associated with a shorter survival rate and worse DFS in CRC patients [57].

Lastly, high expression of the *TM4SF5* gene and protein was detected in GC cells [38,120]. Li et al. [121] showed that overexpression of *TM4SF5* in GC specimens played a crucial role in GC cell proliferation, differentiation, and apoptosis, as evidenced by Gene Ontology analysis and Pathway analysis between cancer and matched normal tissues. They further indicated that the disruption of the TM4SF5-miR-4697-CTD2354A18.1 network might lead to GC development.

The above-mentioned findings indicated that high TM4SF5 expression in PC, CRC, and GC is potentiated to promote cell proliferation, motility, migration, invasion, differentiation, and tumor growth in mice, with reduced apoptosis in cells and poor survival rate among cancer patients.

## 6. The Regulatory Roles and Molecular Mechanisms of TM4SF1, TM4SF4, and TM4SF5 in Cancer Chemoresistance

Chemoresistance is a hallmark of malignant tumors [128], and it is also the major cause of poor survival rates in cancer patients. Ye et al. [16] reported that cell cycle regulation is one of the complex mechanisms of chemoresistance. The study investigated the role of TM4SF1 in LC chemoresistance by using paclitaxel and cisplatin, which are the standard chemotherapy drugs for LC [16,129]. Paclitaxel functions by provoking cell cycle arrest at the G_2_/M phase [130], while cisplatin is a cell-cycle-phase-nonspecific chemotherapy drug. Interestingly, *TM4SF1* silencing using siRNA could regulate the cell cycle by further arresting the G_2_/M phase that subsequently enhanced the sensitivity of A549 cells to both paclitaxel and cisplatin [16].

Additionally, TM4SF1 overexpression reduced apoptosis in MDA-MB-231 cells [50]. It was also reported to exert chemoresistance and promote cell growth, migration, and invasion [16,62]. In contrast, *TM4SF1* silencing induced apoptosis and cell cycle arrest at the G2/M phase in A549 and H1299 cells. It also significantly induced poly-(ADP-ribose) polymerase (PPAR) cleavage and upregulated the expression of apoptotic genes, including *caspase-7*, *caspase-9*, and *caspase-3* in LC [16]. PI3K/AKT/mTOR signaling pathway was also believed to influence the process as siRNA-mediated *TM4SF1* silencing improved cell death and lowered the levels of phosphorylated (p)-AKT, p-mTOR, and p-P70 in BC [50]. By interacting with DDR1, TM4SF1 may regulate the key genes involved in MAPK and AKT pathways, thereby inducing chemoresistance in LC. It has been known that DDR1 is an upstream regulator of the AKT/mTOR pathway [131], a pathway that is involved in the chemoresistance of multiple cancers [132], which in turn interacts with MAPKs [133,134]. Ye et al. [16] also demonstrated that *TM4SF1* silencing could downregulate DDR1 expression and, consequently, inhibit Akt, ERK, and mTOR phosphorylation. Furthermore, the sensitivity of PC cells to gemcitabine was remarkably increased, and the mRNA expression of multidrug resistance (MDR) genes, such as *ABCB1* and *ABCC1*, in AsPC-1, MIAPaCa-2, and PANC-1 cells were decreased after *TM4SF1* silencing. *TM4SF1* silencing also led to the reduction of tumor size in gemcitabine-based treatment in vivo [60]. Therefore, TM4SF1 may serve as a potential biomarker for predicting the treatment response to chemoresistance therapy.

Lewis antigens are tumor-associated carbohydrate antigens that are overexpressed in malignant tumors and are associated with cancer chemoresistance [135]. Overexpression of Lewis(y) antigen stimulated higher production of anti-apoptotic proteins (e.g., Bcl-2 and src-xL) and reduced the expression of the pro-apoptotic proteins (e.g., Bax and caspase-3), causing the inhibition of cell apoptosis and promotion of chemoresistance occurrence. Intriguingly, blocking Lewis(y) antigen could significantly reverse the effects [17]. Liu et al. [17] revealed that the mRNA levels of *TM4SF4* were significantly increased in Lewis(y) highly expressed chemoresistant OC, suggesting that Lewis(y) causes cancer chemoresistance due to apoptosis inhibition.

Other than the above findings, TM4SF5-mediated EMT may have an important function in chemoresistance [41]. Suppression of TM4SF5 in gefitinib-resistant cells via T790M EGFR mutation caused the cells to become more sensitive toward gefitinib and displayed more epithelial-like instead of mesenchymal-like cell characteristics. EMT mediated by TM4SF5 and cell surface regulation of EGFR, mesenchymal-epithelial transition factor (c-MET), and p27Kip1 activity may significantly cause LC gefitinib resistance [136]. Additionally, TM4SF5 may also induce chemoresistance and cancer fibrosis by interacting with integrins α2, α5, β1, and EGFR [41,136]. For instance, it has been reported that TGFβ1-mediated Smad actions activate EGFR to express TM4SF5, resulting in EMT activation and the formation of murine liver fibrosis [41,117]. Through interaction with integrin α2, TM4SF5 has been demonstrated to regulate actin remodeling in Cos7 fibroblasts. Thus, crosstalks between TM4SF5 and other membrane receptors, such as integrins and growth factor receptors, are thus likely to play a role in regulating EMT to mediate chemoresistance and cancer fibrosis [136].

**Figure 5:**
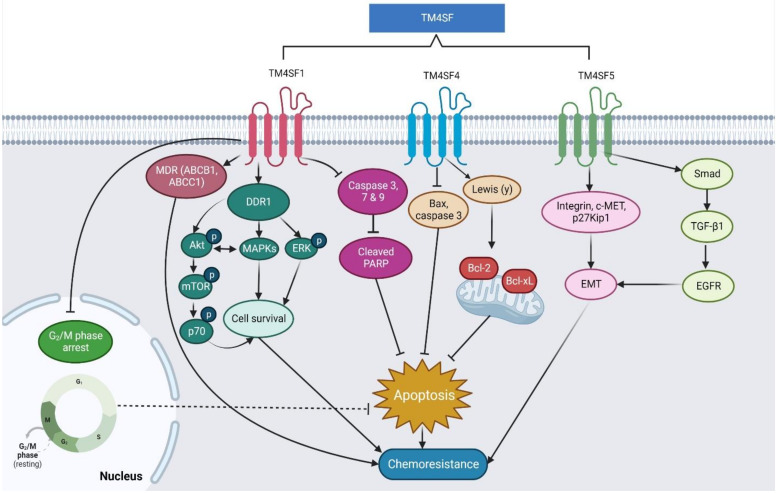
Proposed mechanism underlying the roles of TM4SF1, TM4SF4, and TM4SF5 toward cancer chemoresistance.

The summative mechanisms underlying the TM4SF activity in chemoresistance are illustrated in Figure 5, while its roles in different cancers and the associated molecular mechanisms are summarized in Table 4.

## 7. The Current Use of Antibodies in Targeting TM4SF as a Potential Cancer Treatment

For the past 20 years, monoclonal antibody-based treatment has been established as one of the most successful therapeutic approaches to treating cancers [137]. Apart from surgery, chemotherapy, and radiation, this treatment has been considered the main element of cancer therapy because it possesses diverse clinically relevant mechanisms, specifically targeting and promoting long-lasting anti-tumor immune response [138]. In this review, several preclinical studies described above have used or developed different antibodies to specifically target the expression of TM4SF1, TM4SF4, or TM4SF5, followed by observing the effects in reversing their regulatory roles in cancer phenotypes and chemoresistance. Thus, all anti-TM4SF antibody-related treatment data are described and discussed here to evaluate their potential, efficacy, and current use.

A recent study showed that anti-TM4SF1 antibody-drug conjugates could be a promising therapeutic agent to combat cancer cells and vasculature in the lung, pancreas, prostate, and colon [59,62,97]. Apart from these results, an earlier report successfully demonstrated that a mouse anti-human TM4SF1 monoclonal (IgG1) antibody (8G4) was able to destroy human components of the vascular network and kill human PC-3 cells via the antibody-dependent cell-mediated cytotoxicity (ADCC) mechanism [59]. Meanwhile, treatment with an anti-TM4SF4 antibody was shown to suppress insulin growth factor-1 (IGF1)/IGF1R signaling pathway-activated growth, migration, and invasion in both radiation-resistant A549 and Calu 3 cells [48].

It has been identified that the effects of TM4SF5 on cell migration and tumorigenesis are associated with integrins α2, α5, and β1, and EGFR or CD44, probably occurring via its EC2 [115,136]. For instance, blocking the EC2 domain using TSAHC, an anti-TM4SF5 compound, or by introducing point mutations at N-glycosylation residues within EC2 could inhibit TM4SF5-mediated growth and promote the loss of contact inhibition and invasion of tumor cells [109], thus highlighting the critical role of the EC2 domain in TM4SF5-mediated functions. Furthermore, Kwon, Choi [55] also evaluated the effect of the anti-TM4SF5 antibody on HCC cell migration and invasion. They found that the migratory capacity of HCC Huh-7 cells in a wounded area was significantly reduced. Withal, the treatment of HCC with anti-TM4SF5 monoclonal antibody inhibited tumor growth in a syngeneic BNL-HCC cells-transplanted mouse model.

In addition, the anti-TM4SF5 monoclonal antibody was also found to be implicated in HCC cells by modulating cell signaling, reducing cell motility, enhancing E-cadherin expression, altering p27^kip1^ localization, and increasing RhoA activity. Additionally, it also could significantly attenuate tumor growth in both mouse xenograft and mouse syngeneic transplanted HCC models [55]. Apart from these, TM4SF5 plays a critical role in liver fibrosis, which is a hallmark of cirrhosis that stimulates HCC development [96,139]. For instance, Ahn, Ryu [23] demonstrated that Ab27 and Ab79, which are chimeric antibodies binding to E2 of TM4SF5, prevented the development of fibrotic phenotype in a carbon tetrachloride (CCl4)-mediated mouse liver fibrosis model. This finding indicated that these anti-fibrosis antibodies could prevent HCC progression. Additionally, they also showed that Ab27 was more effective than Ab79. The former significantly inhibited HCC cell invasion and proliferation and decreased tumor growth in HCC xenograft nude mice model via TM4SF5 neutralization [23].

Interestingly, immunization with the TM4SF5 peptide vaccine suppressed the tumor growth in the allograft mouse model injected with TM4SF5-expressing PC cells [58]. Treatment with the anti-TM4SF5 antibody could inhibit the expression of EMT markers (i.e., Vimentin and E-cadherin) and reduce the proliferation and motility of PC cells that endogenously expressed TM4SF5 [140]. Furthermore, TM4SF5 could significantly induce the proliferation and motility of mouse PC cells. The use of anti-hTM4SF5 monoclonal antibody notably diminished PC cell growth and motility and modulated the expression of EMT markers, such as vimentin and E-cadherin [58]. 

Kwon, Choi [55] demonstrated that the anti-TM4SF5 antibody developed through the immunization with TM4SF5 peptide-CpG-DNA-liposome complex markedly inhibited CRC cell growth. The robust production of TM4SF5-specific antibodies was then induced by a challenge with CRC cells. The tumor growth was significantly suppressed in the peptide vaccine targeting TM4SF5 mice, thus showing a prophylactic effect against CRC development in a mouse model [141].

Apart from that, a novel monoclonal antibody, mEC2-CF, was developed to target a cyclic epitope of TM4SF5, and its reactivity to TM4SF5 in CRC cells and tissues was also evaluated. Upon binding to the membrane-associated TM4SF5, the antibody was internalized into the cytosol of CRC cells. These observations suggest that this antibody may be useful for therapeutic cancer treatments, at least in CRC [57].

## 8. Conclusions and Future Perspectives

This review provided an in-depth insight into the molecular expression and regulatory roles of TM4SF1, TM4SF4, and TM4SF5 in cancer progression, metastasis, angiogenesis, and chemoresistance by identifying the mechanistic pathways and key molecules involved. It also discussed the available and current use of existing preclinical antibodies targeting TM4SF1, TM4SF4, and TM4SF5 to combat various cancers. The regulatory roles of TM4SF1, TM4SF4, and TM4SF5 in the described cancer phenotypes and chemoresistance have been intensively evaluated in the past ten years as compared to other TM4SF members under the transmembrane 4 L6 domain family. Thus, the investigation of TM4SF members as potential molecular targets, which could lead to a novel drug discovery to combat cancer, is considered new. Most studies have indicated that TM4SF1, TM4SF4, and TM4SF5 are highly expressed in the cells and tissues of different cancers with low OS, indicating their potential diagnostic and prognostic values. Of the three members, TM4SF1 has been the most extensively studied. However, studies using TM4SF1 in PC, GC, and HCC as potential prognostic markers are still controversial. It should be approached with caution, considering the specific type of sample and tissue chosen.

The three TM4SF members are involved in regulating cell proliferation, migration, invasion, EMT, angiogenesis, and chemoresistance of various cancers, as evidenced by preclinical studies. Intriguingly, their modulation in the proliferation, migration, and invasion of cancer cells might involve JAK/STAT3 signaling pathway, coupled with DDR1, Rock-independent Rho GTPase, or Akt/PI3K/mTOR pathways. Additionally, their mediation in chemoresistance is mainly achieved via DDR1 coupled with Akt/ERK/mTOR signaling or associated with a high expression of Lewis (y) antigen. Furthermore, the downregulation of TM4SF1, TM4SF4, and TM4SF5 expression in different cancer types, either using gene silencing or anti-TM4SF antibody, could reverse the observed effects on cancer phenotypes and progression. In the use of antibody-mediated preclinical cancer treatment, more studies have investigated its potential use against TM4SF5 as compared to TM4SF1 and TM4SF4. In addition, no such anti-TM4SF study on chemoresistance has been reported yet and thus deserves further investigations. 

Moreover, the generation of ROS is widely known to be an indicator of cancer progression [142]. Elevated ROS caused by TM4SF1 downregulation can induce cell cycle arrest and apoptosis in human BCa [77]. Additionally, a few phytochemicals, such as curcumin and epigallocatechin gallate, have been shown to control cancer progression by exhibiting antioxidant and prooxidant properties [143,144]. Hence, the involvement of ROS in TM4SF-mediated cancer progression, metastasis, and chemoresistance deserves further investigation. Given that miRNAs are contributed to TM4SF1- and TM4SF5-mediated PC and GC phenotypes, thus it indicates that it is worth investigating further the interactions between miRNAs and TM4SF as well as non-coding RNAs, particularly long non-coding RNAs that have been reported to overexpress in various cancers to hijack their progression, metastasis, and response to chemoresistance [4,9,21]. Additionally, more in vitro and in vivo studies investigating the regulatory roles of these three TM4SF members in various cancers, as well as determining the stability and efficacy of the candidate anti-TM4SF antibodies in detail, are required to expand its role as a potential therapeutic molecular target. Given the significant impact of using anti-TM4SF antibodies, the potential use and therapeutic effects of aptamer, which is considered a chemical antibody and widely accepted as a safe replacement for antibodies, as well as the nanotechnology-assisted delivery of anti-TM4SF, are deemed worthy of investigation in the future, to target TM4SF effectively [145]. This review collectively suggests that TM4SF1, TM4SF4, and TM4SF5 are potential and emerging molecular targets, and targeting them in the described cancers could result in the development of personalized medicine.

## Figures and Tables

**Table 1:** Molecular expression, regulatory roles, biomolecular interactions, and the underlying mechanism of actions of TM4SF1 in different cancers against cancer phenotypes and progression.

Cancer Types	Test Model (In Vitro/In Vivo/Clinical)	Metabolic Responses/Mechanisms	Reference
Prostate	In vitro (PC-3, DU145, LNCaP and VCaP)	Messenger ribonucleic acid (mRNA) expression and protein levels of TM4SF1 were significantly higher compared to benign prostatic hyperplasia (BPH) specimens. Activation of TM4SF1 induced proliferation and migration of cancer cells via the androgen receptor (AR) pathway. Inhibition of TM4SF1 protein expression revoked the migration ability of metastatic PC-3 cancer cells and non-prostatic (HeLa) cancer cells. High TM4SF1 expression in PRC significantly activated extracellular signal-regulated kinase (ERK1/2) signaling pathway, increased EMT-related protein (e.g., E-cadherin, V-cadherin, and Vimentin), and enhanced invasion, migration, and proliferation of PRC cells.	[27,70,71,72]
	In vivo (Nude mice)	TM4SF1 highly expressing in PC3 PRC cells were implanted subcutaneously into nude mice along with human endothelial colony-forming cells (ECFC)/human mesenchymal stem cells (MSC) cells and greatly increased the vascularity of Matrigel plugs.	[59]
	Clinical (Human prostate tumor tissue)	*TM4SF1* mRNA level in prostate tumor samples was significantly higher than in non-cancerous prostate glands resected from patients with BPH.	[27]
Pancreas	In vitro (AsPC-1, MIA PaCa-2, PANC-1, SW1990 and BxPc-3 cells)	TM4SF1 was overexpressed in the PC cells, and its silencing reduced the migration and invasion of cancer cells, as well as downregulated the expression and activity of MMP-2 and MMP-9. Suppression of TM4SF1 reduced the expression levels of DDR1 and the formation of invadopodia in PANC-1 and AsPC-1 cells, thus inhibiting PC cell migration and invasion. has-miR-141 microRNA (miRNA) was reported to inhibit TM4SF1 expression by directly targeting the binding site in its 3′UTR region; thus, PC cell migration and invasion were attenuated. However, no effect of TM4SF1 on the proliferation and apoptosis of PC cells was observed.	[31,49,67]
	Clinical (Human PC tissue)	*TM4SF1* mRNA levels were higher in PC tissues, and the expression was positively correlated with *DDR1* mRNA, which was linked to poor prognosis and shorter survival times. However, other studies showed that low levels of TM4SF1 in PC tissues had higher tumor grade, advanced clinical stages, and shorter survival times. Local spread was also more common with the low TM4SF1 expression group.	[30,67]
Gastric	In vitro (MGC803 and MKN45 cells)	TM4SF1 was highly expressed in human gastric carcinoma (MGC803 and MKN45). *TM4SF1* silencing significantly decreased *B-cell lymphoma 2 (Bcl2)* mRNA expression while increasing *caspase-3* and *Bcl-2-associated X protein (Bax)* expression levels. TM4SF1 downregulation promoted apoptosis and decreased proliferation, migration, and invasion of MGC803 and MKN45 cells.	[52]
	Clinical (Gastric mucosa tissues)	Low TM4SF1 expression was associated with tumor carcinogenesis, progression, and invasion, as well as a poor overall survival (OS) rate.	[73,74]
Breast	In vitro (MDA-MB-231 cells)	TM4SF1 overexpression induced migration and reduced apoptosis in MDA-MB-231 BC cells. *TM4SF1* silencing downregulated the expression of p-mTOR, p-P70, and p-AKT, followed by the inhibition of cancer cell migration.	[50]
	In vivo (Syngeneic BALB/c mice)	Through its non-canonical association with collagen receptor tyrosine kinase DDR1 via syntenin 2, protein kinase C (PKC), JAK2, and STAT3 signaling pathways, TM4SF1 acted as a strong mediator for metastatic reactivation of BC. Later, this pathway activated Sry-related HMG box 2 (SOX2) and *Nanog* Homeobox (NANOG), resulting in metastatic reactivation in the lungs, bones, and brain.	[75]
	Clinical (BC tumor)	1. Low estrogen receptor (ER), low progesterone receptor (PR), and low human epidermal growth factor receptor 2 (HER2) expression were linked to high TM4SF1 expression in triple-negative breast cancer (TNBC).2. Disease-free survival (DFS) and OS were expected to be shorter in these patients.	[76]
Ovarian	In vitro (HO8910PM and SKOV3 cells)	*TM4SF1* silencing did not affect growth, proliferation, or cell cycle distribution of HO8910PM and SKOV3 cells, but their migration and invasion capacities were significantly suppressed.	[13]
	In vivo (Nude mice)	The growth of xenograft tumors in nude mice was inhibited by *TM4SF1* downregulation.	[13]
	Clinical (Epithelial OC tissues)	TM4SF1 had a higher expression in epithelial OC tissues than benign ovarian tumor tissues and normal ovarian epithelial tissues. The late stage of OC has significantly higher protein expression than the early stage.	[13]
Hepatocellular	In vitro (HepG2 and HUVEC cells)	Overexpressed TM4SF1 reduced apoptosis and promoted migration of HCC cells. TM4SF1 regulated apoptosis and migration-related genes, such as *caspase-3*, *caspase-9*, *MMP-2*, *MMP-9,* and *VEGF*, which are associated with liver tumor growth and progression. TM4SF1 was highly expressed in the vascular endothelium cells of human cancers and regulated angiogenesis. *TM4SF1* silencing inhibited filopodia formation, cell mobility, and cytokinesis and rendered EC senescent. 5. Integrin-alpha5 and integrin-beta1 subunits interacted constitutively with TM4SF1.6. AlphaV, beta3, and beta5 (often associated with angiogenesis) interacted with TM4SF1 only after stimulation of VEGF-A or thrombin.7. TM4SF1 interacted with integrins to mediate endothelium cell migration and enhanced cell–cell interaction.	[66,69]
	In vivo (Foxn1^−/−^ nude mice)	In a mouse model, TM4SF1 increased tumor development and metastasis, but these effects were reversed after *TM4SF1* silencing.	[69]
Bladder	In vitro (T24, EJ and UM-UC-3 cells)	TM4SF1 silencing using short hairpin RNA (shRNA) inhibited bladder cancer (BCa) cell proliferation. TM4SF1 silencing could induce cell cycle arrest, Reactive Oxygen Species (ROS) generation, catalase, and superoxide dismutase 2 (SOD2) upregulation, leading to oxidative stress-induced apoptosis. These events may be mediated by a Peroxisome proliferator-activated receptor γ-Sirtuin 1 (PPARγ-SIRT1) negative feedback loop.	[77]
	In vivo (NOD/SCID xenotransplanted tumor mice)	TM4SF1 silencing using shRNA significantly inhibited tumor growth.	[77]
	Clinical (Human muscle invasive bladder cancer (MIBC) tissues)	TM4SF1 was highly expressed in human MIBC tissues. It was significantly correlated with Tumor stage (T stage), The Tumor Node Metastasis (TNM) stage, lymph node metastasis status, and a lower OS rate. High levels of TM4SF1 in tumor specimens may indicate a high risk of BCa and a poor prognosis for BCa patients.	[77]

**Table 2:** Molecular expression, regulatory roles, biomolecular interactions, and the underlying mechanism of actions of TM4SF4 against cancer phenotypes and progression.

Cancer Types	Test Model (In Vitro/In Vivo/Clinical)	Metabolic Responses/Mechanisms	Reference
Lung	In vitro (HCC-1833, A549 and Calu-3 cells)	TM4SF4 was highly expressed in HCC-1833, Calu-3, and A549 LC cells. *TM4SF4* knockdown by siRNA or shRNA caused cell cycle arrest and consequently inhibited cell growth. High TM4SF4 expression in radiation-resistant LC increased the activation of PI3K, AKT, NF-κB, PTEN, insulin growth factor-1 receptor (IGF1R), and epidermal growth factor receptor (EGFR). Its overexpression activated cell growth, migration, and invasion. TM4SF4 overexpression also enhanced OPN secretion, which activated CD44 or integrin signaling, and maintained EMT-associated cancer stem-like cell (CSC) features. Through the TM4SF4-mediated focal adhesion kinase (FAK)/STAT3 signaling pathway overexpressed OPN increased self-renewal and metastatic properties of cancer cells.	[48,53,100]
	In vivo (Athymic BALB/c nude mice)	Immunohistochemical staining of xenografted nude mice tissues showed the tumorigenic activity of TM4SF4 in LC.	[48]
	Clinical (LC tissue)	*TM4SF4* was shown as one of the top five genes significantly expressed in alectinib-resistant LC patients, and it was strongly associated with nuclear division, mitosis, and cell cycle. *TM4SF4* mRNA was sorted as an outlier gene from the Cancer Cell Line Encyclopedia (CCLE) and The Cancer Genome Atlas (TCGA) data with high absolute expression levels relative to other genes. Five of 119 LC cases had scored high *TM4SF4* expression through tissue microarray analysis. However, it was difficult to determine the relevance of clinicopathological features based on *TM4SF4* expression as the outlier sample sizes were too small.	[100,101]
Colorectal	Clinical (CRC tissue and CRC tumor buds)	CRC metastasis was significantly associated with TM4SF4 overexpression, indicating the potential role of TM4SF4 as a biomarker for the advanced stage of CRC. High expression of TM4SF4 in tumor specimens had poor CRC prognosis, reduced survival potential, and correlated with cancer development. TM4SF4 was involved in EMT process mediated by TGFβ/Snail and TNFα/NFκB pathway.	[32,102]
Hepatocellular	In vitro (QGY-7701, SMMC-7721 and BEL-7404 cell)	TM4SF4 overexpression in promoted HCC cell proliferation and colony formation, and reduction of its level reversed the observed effects. *TM4SF4* silencing using adenovirus-delivered siRNA targeting TM4SF4 (AdSiTM4SF4) in HCC cells significantly inhibited the endogenous TM4SF4 expression and effectively reduced cell growth and colony formation.	[54,103]
	In vivo (Xenograft tumor model nude mice)	Injection of *TM4SF4* siRNA into xenograft nude mice significantly reduced tumor growth and size.	[54]
	Clinical (HCC tissue)	*TM4SF4* mRNA and protein levels were highly expressed in HCC specimens as compared to non-tumor tissues. Its localization was more abundant on the plasma membrane, less in the cytoplasm, and had none in the nucleus. The expression of TM4SF4 protein in HCC was correlated to tumor growth, as higher levels of TM4SF4 were found in the early stages of the disease.	[54,103]

**Table 3:** Molecular expression, regulatory roles, biomolecular interactions of TM4SF5 in different cancers, and the underlying mechanism of actions against cancer phenotypes and progression.

Cancer Types	Test Model (In Vitro/In Vivo/Clinical)	Metabolic Responses/Mechanisms	Reference
Hepatocellular	In vitro (SNU449 and Huh7 cells)	TM4SF5 improved cytosolic stability and morphological elongation by increasing the expression of p27Kip1 (a cyclin-dependent kinase (CDK) inhibitor/tumor suppressor) through RhoA modulation. *TM4SF5* silencing abolished the expression of p27Kip1 and reconstituting E-cadherin, which was involved in EMT process in Snail1-independent manner. TM4SF5 increased FAK Tyr577 phosphorylation associated with FAK, Rho GTPase-activating protein, and cellular Src (c-Src) phosphorylation, causing the inactivation of protein RhoA. TM4SF5 also induced self-renewal and other properties of circulating tumor cells via the interaction with CD44. Due to the loss of contact inhibition, TM4SF5-mediated RhoA inactivation promoted EMT, resulting in tumor cell migration, invasion, and proliferation. TM4SF5 overexpression was also correlated with VEGF expression and vessel formation in HCC SNU449 cells and clinical HCC samples, respectively.	[38,112,113,114,115]
	In vivo (BALB/c-n/n mice)	TM4SF5 overexpression facilitated migration, invadopodia formation, MMP activation, invasion, and eventually lung metastasis suppression, whereas *TM4SF5* silencing with shRNA had the opposite effect.	[116]
	Clinical (Tumor liver tissues)	TM4SF5 was found to enhance cytosolic stabilization and morphological elongation by increasing the expression of p27^Kip1^ via RhoA modulation. The levels of cyclin D1 were higher in TM4SF5-positive HCC tissues, whereas the levels of p16 and p18 CDK inhibitors were lower. EGFR phosphorylation as well as Proline Extensin-like Receptor Kinase 1 (pErk1)/2, β-catenin, and myc levels had no correlation with cyclin D1 levels. TM4SF5 overexpression also correlated with VEGF expression and vessel formation in HCC SNU449 cells and clinical HCC samples, respectively, and required the regulation of integrin-α5, c-Src, and STAT3. Tumor tissue from HCC patients had higher levels of TM4SF5 and *Suppressor of Mothers Against Decapentaplegic 2* (Smad2)/3 phosphorylation, implying a possible link between TGF1 signaling and TM4SF5 expression in liver carcinogenesis.	[38,112,115,117]
Esophageal	In vitro (KYSE150 cells)	Downregulation of TM4SF5 reduced cell proliferation, metastasis, and invasion. The activity of TM4SF5 involved the interferent of integrin β1 and reduced the cell invasion on laminin.	[110]
Pancreas	In vitro (PANC02 cells)	*TM4SF5*-transfected PANC02 cells markedly increased cell proliferation and motility.	[58]
	In vivo (C57BL/6 allograft mice model)	*TM4SF5*-transfected PANC02 cells markedly increased tumor mass in mice.	[58]
	Clinical (PC tissue)	Overexpression of *TM4SF5* mRNA was detected in examined PC tissues.	[39]
Colorectal	In vitro (CT-26, LoVo, and SW480 cells)	TM4SF5 expression detected in a mouse CRC cell line. *TM4SF5* silencing in CRC cells significantly reduced cell proliferation, migration, and invasion.	[118,119]
	Clinical (CRC tissues)	Overexpression of *TM4SF5* mRNA in CRC patients was linked to a lower survival rate and a worse DFS rate.	[23,57]
Gastric	In vitro (SNU601 cells)	*TM4SF5* gene and protein were highly expressed in GC cells.	[38,120]
	Clinical (GC tissues)	TM4SF5 played a crucial role in GC cell proliferation, differentiation, and apoptosis. Disruption TM4SF5-miR-4697-CTD2354A18.1 network may lead to GC development.	[39,121]

**Table 4:** Regulatory roles of TM4SF1, TM4SF4, and TM4SF5, and the underlying mechanism of actions against chemoresistance in different.

TM4SF	Cancer Types	Test Model (In Vitro/In Vivo)	Metabolic Responses/Mechanisms	Reference
TM4SF1	Lung	In vitro (A549 and H1299 cells)	The percentage of cells in the G_2_/M phase in the siRNA-*TM4SF1* transfected group was greater than the control group, and the cell population in the S phase was also greater. siRNA-mediated *TM4SF1* silencing increased the sensitivity of A549 and H1299 cells to paclitaxel and cisplatin as compared to the controls. *TM4SF1* silencing induced cell apoptosis in both A549 and H1299 cells as compared to control. It significantly induced PARP cleavage and upregulated *caspase-7*, *caspase-9*, and *caspase-3*. TM4SF1 also regulated DDR1 and the phosphorylation of its downstream targets, including AKT, ERK, and mTOR. *TM4SF1* silencing downregulated DDR1/AKT pathway after mTOR inhibitor treatment.	[16]
	Breast	In vitro (MDA-MB-231 cells)	Overexpression of TM4SF1 was negatively correlated with MDA-MB-231 cell apoptosis. As pcDNA-TM4SF1 transfected cells were compared to control, apoptosis was significantly reduced. *TM4SF1* silencing using siRNA improved cell apoptosis and lowered the expressions of phosphorylated (p)-AKT, p-mTOR, and p-P70. PI3K/AKT/mTOR signal pathway influenced cell process.	[50]
	Pancreatic	In vitro (AsPC-1, MIA PaCa-2 and PANC-1 cell)	*TM4SF1* silencing in PC cells increased its sensitivity to gemcitabine and downregulated the mRNA expression of MDR genes (i.e., *ABCB1* and *ABCC1*).	[60]
		In vivo (Athymic nude nu/nu mice)	Stably *TM4SF1* silencing in MIA PaCa-2 cells was developed in immuno-deficient mice and led to reduction of tumor size after treatment with gemcitabine.	[60]
TM4SF4	Ovarian	In vitro (RMG-I-H, RMG-I, COC1/DDP and COC1 cells)	Lewis(y) highly expressed chemoresistant OC had significantly higher mRNA levels of *TM4SF4*, suggesting that Lewis(y) caused cancer chemoresistance by inhibiting apoptosis.	[17]
TM4SF5	Lung	In vitro (Gefitinib-sensitive cells; HCC827, Gefitinib-resistant cells; NCI-H358)	Gefitinib-resistant cells became more sensitive and displayed more epithelial-like instead of mesenchymal-like cell characteristics. EMT mediated by TM4SF5 and cell surface regulation of EGFR, c-MET, and p27Kip1 activity may be significant in causing resistance.	[136]
	Liver	In vitro (SNU449)	TM4SF5 bound with integrin α5 and was more efficiently retained on the surface of TM4SF5-expressing hepatocytes than on the surface of TM4SF5-deficient cells.	[115]
		In vivonihao(TM4SF5-overexpressing transgenic mice, zebrafish)	TGFβ1-mediated Smad actions activated EGFR to express TM4SF5, which resulted in EMT activation and formation of murine liver fibrosis. In zebrafish, TM4SF5 suppression altered the expression and localization of integrin α5, resulting in abnormal muscle fiber development, which is required for somite boundary maintenance. TM4SF5 expression is thus important in the development of zebrafish muscles, which may be mediated by EMT, in addition to liver fibrosis and tumorigenesis.	[41,117]

## Data Availability

Not applicable.
